# Hospital Nurses in Asylum Service

**Published:** 1897-02-20

**Authors:** 


### EDITOR'S LETTER-BOX.

[Our Correspondents are reminded that prolixity is a great bar to publication, and that brevity of style and conciseness of statement greatly facilitate early insertion.]

### HOSPITAL NURSES IN ASYLUM SERVICE.

Dr. George Robertson, Perth District Asylum, writes: As I do not profess to replace "ignorance and cocksureness with common sense and usefulness," either with or without the aid of an electric machine, such psychological transformations exist only in the mind of "M. D." I stated that hospital nurses are first-class material for making mental nurses of, and I objected to Dr. Urquhart's remarks as they conveyed the contrary impression. Unfortunately for "M. D.'s" arguments, Dr. Urquhart is also among those "at variance with the opinion of ripe experience," for he informs me it would be the irony of fate to regard him as objecting to hospital nurses in asylum service. My remarks regarding the "hysterical theory," which have served as a butt for "M. D.'s" wit, were, I hope, regarded as a mere *façon de parler* by everyone else, and the objection is regarded by Dr. Urquhart himself as being so slender as not to carry sufficient weight to influence his practice, which has been to encourage hospital nurses. These suggestions may be neither new nor any advance, but I feel confident that if they were more universally adopted the regrettable differences that exist between mental nurses and hospital nurses would disappear to a very large extent, and that the weaknesses in the asylum service pointed out by an independent critic like the editor of The Hospital would soon be a thing of the past.

